# Non-inferiority randomised phase 3 trial comparing two radiation schedules (single vs. five fractions) in malignant spinal cord compression

**DOI:** 10.1038/s41416-020-0768-z

**Published:** 2020-03-11

**Authors:** Pierre G. Thirion, Mary T. Dunne, Paul J. Kelly, Aileen Flavin, Joe M. O’Sullivan, Dayle Hacking, Wojciech Sasiadek, Cormac Small, Maeve M. Pomeroy, Joseph Martin, Orla McArdle, Imelda Parker, Lydia S. O’Sullivan, Aoife M. Shannon, Angela Clayton-Lea, Conor D. Collins, Michael R. Stevenson, Alberto Alvarez-Iglesias, John G. Armstrong, Michael Moriarty

**Affiliations:** 1 Cancer Trials Ireland (formerly All-Ireland Cooperative Oncology Research Group (ICORG)) and St Luke’s Radiation Oncology Network (SLRON), Dublin, Ireland; 2 SLRON, Dublin, Ireland; 3 Cork University Hospital, Cork, Ireland; 4 Belfast City Hospital, Dublin, Ireland; 5 UPMC Whitfield Cancer Centre, Waterford, Ireland; 6 University of Pittsburgh Medical Center, Whitfield Cancer Centre, Waterford, Ireland; 7 Galway University Hospital, Galway, Ireland; 8 Cancer Trials Ireland, Dublin, Ireland; 9 Statistics and Data Management Office for ICORG, Clinical Research Support Centre, Belfast, Ireland; 10 The HRB Clinical Research Facility, Galway, Ireland

**Keywords:** Radiotherapy, Metastasis

## Abstract

**Background:**

The optimal EBRT schedule for MSCC is undetermined. Our aim was to determine whether a single fraction (SF) was non-inferior to five daily fractions (5Fx), for functional motor outcome.

**Methods:**

Patients not proceeding with surgical decompression in this multicentre non-inferiority, Phase 3 trial were randomised to 10 Gy/SF or 20 Gy/5Fx. A change in mobility from baseline to 5 weeks for each patient, was evaluated by a Modified Tomita score: 1 = ‘Walk unaided’, 2 = ‘With walking aid’ and 3 = ‘Bed-bound’. The margin used to establish non-inferiority was a detrimental change of −0.4 in the mean difference between arms.

**Results:**

One-hundred and twelve eligible patients were enrolled. Seventy-three patients aged 30–87 were evaluated for the primary analysis. The 95% CI for the difference in the mean change in mobility scores between arms was −0.12 to 0.6. Since −0.4 is not included in the interval, there is evidence that 10 Gy/SF is non-inferior to 20 Gy/5Fx. One grade 3 AE was reported in the 5Fx arm. Twelve (26%) patients in the 5Fx arm had a Grade 2–3 AE compared with six (11%) patients in the SF arm (*p* = 0.093).

**Conclusion:**

For mobility preservation, one 10-Gy fraction is non-inferior to 20 Gy in five fractions, in patients with MSCC not proceeding with surgical decompression.

**Clinical Trial Registration:**

Cancer Trials Ireland ICORG 05-03; NCT00968643; EU-20952.

## Background

Malignant epidural spinal cord compression (MSCC) is a common cancer-related neurological complication, associated with poor functional outcome, a deterioration in the quality of life and short survival. The lifetime MSCC incidence for cancer patients is between 1 and 10%,^1–3^ with a reported median time of less than a year after initial diagnosis.^4^ Large retrospective studies reported a median survival time of 3–6 months for patients with MSCC,^2,5^ the main favourable prognostic factors being radiosensitive tumours, other than lung primary, isolated spinal metastasis, no associated visceral or brain metastasis and pre-treatment-preserved mobility.^6,7^

Standard emergency therapies include high-dose steroids in combination with either surgery followed by external beam radiation therapy (EBRT), or primary EBRT.^2^ The therapeutic goals are stabilisation or improvement of neurological function, and pain relief. Despite a randomised trial by Patchell and colleagues, showing superior functional outcome with combined surgery and EBRT over primary EBRT^8^—with a reported 84% post-treatment ambulatory rate—most patients continue to be treated by primary EBRT as it can be difficult to justify the risks of surgery for this patient category.^2,8^ In addition, the Patchell surgical trial results may not be applicable to most patients because their selection criteria excluded what is likely the majority of spinal cord compression patients. For example, they had to have an expected survival of >3 months, could only have one level of cord compression, no brain metastases, no paraplegia >48 h and acceptable medical status. The accrual period of 10 years may reflect the restrictive selection criteria for surgical intervention. There is debate regarding the optimal primary EBRT radiation schedule, with various radiation schedules used prior to the start of this trial, ranging from mildly hypofractionated (10–15 fractions) to single-fraction (SF) schedules.^9–19^

For patients with a short life expectancy, prolonged fractionation schedules may take up a substantial proportion of their remaining life, making a SF schedule an attractive practical approach, provided that similar therapeutic outcome is demonstrated.

A commonly used schedule of 20 Gray in five daily fractions (20 Gy/5Fx) has been recommended for patients with an estimated life expectancy of less than 6 months.^17,18^ Despite multiple studies reporting similar outcomes with SF RT,^9,10,14,16–18^ worldwide surveys of patterns of practice show consistent underuse of SF for bone metastases.^5^

### Purpose/objective

The aim of the Cancer Trials Ireland (formerly All Ireland Cooperative Oncology Research Group) ICORG 05-03 study was to determine if a 10 Gy/SF of EBRT is not inferior to the commonly used radiation schedule of 20 Gy/5Fx, in terms of functional motor outcome, for the treatment of MSCC patients not proceeding with surgical decompression. The results were presented at the American Society for Radiation Oncology 2014 meeting.^20^ This paper presents the full report of the study based on the analysis of eligible patients with available data at baseline and at the 5-week follow-up.

## Methods

### Study design

The trial was conducted across five Irish sites (Belfast, Cork, Dublin, Galway and Waterford). Patients were randomly assigned to one of two treatment arms in a 1:1 ratio, to receive EBRT delivering either an experimental 10 Gy/SF, or a 20 Gy/5Fx standard fractionated radiation schedule. An Independent Data Monitoring Committee (IDMC) reviewed unblinded data for patient safety, and found no safety concerns with the trial intervention. There were no interim analyses for efficacy or futility. The primary outcome was the change in mobility status between baseline and the 5-week follow-up, evaluated by an in-house modified Tomita mobility scale that had three possible scores: 1 = ‘Unaided’, 2 = ‘With walking aid’ and 3 = ‘Bed-bound’. The scale was modified to allow for telephone follow-up.

A detrimental difference of 0.4 between the two arms in the mobility-scale change between the baseline and 5-week assessment, was deemed unacceptable. An interim analysis in March 2011 of 47 evaluable patients found a residual standard deviation of 0.7, which was used in the sample size calculation. For this non-inferiority study to have a power of 80% for a test using a one-sided 95% confidence limit, that the mean difference in the mobility change between the arms would not include −0.4, required 38 evaluable patients in each arm, 76 evaluable patients overall. Evaluable patients were those with a documented mobility status at 5 weeks. A sample size of 126 patients was estimated as being necessary, given an anticipated early death-related attrition of 40%.

### Participants

#### Eligibility criteria

The inclusion criteria were (1) age ≥18 years, (2) magnetic resonance imaging (MRI)-documented symptomatic MSCC/cauda equina syndrome (whole-spine MRI required), (3) histologically proven malignancy (excluding leukaemia, myeloma, lymphoma, germ cell tumours and primary spinal bone tumours), (4) Karnofsky Performance Status (KPS) ≥30% and (5) written informed consent. Patients with ≤2 compression levels were eligible. For the purpose of the trial, the diagnosis of symptomatic MSCC or cauda equina syndrome was based on a combination of clinical symptoms and MRI-based radiological criteria. The symptoms could be MSCC-level-related pain and/or neurological symptoms. MRI-based definition of MSCC was an epidural mass, touching, displacing, indenting the spinal cord or leading to complete loss of definition of the spinal cord or cauda equina.

The exclusion criteria were (1) previous irradiation of any spinal segment to be included within the RT volume for the treatment of the MSCC, (2) isolated bone metastasis with controlled primary site, (3) patients deemed suitable for neurosurgical intervention or (4) those with a medical or psychiatric condition, which in the opinion of the investigator, contraindicates participation.

#### Randomisation

Patients were randomly assigned, using simple randomisation procedures (computerised random numbers), to one of two arms. The allocation sequence was concealed from the investigator in sequentially numbered, opaque sealed envelopes, opened only after the enrolled participants completed all baseline assessments. The sealed randomisation envelopes were prepared centrally for each participating centre prior to enrolling patients, each of the participating hospitals having their own series of sequential envelopes, and the randomisation arms being statistically pre-determined from a randomisation master list. The study could not be blinded, as this is not practical in the case of a radiation study addressing a fractionation question.

#### Treatment

The steroids, dose schedule and the EBRT technique (field arrangement, beam type and energy) were left to institutional practice and physician preferences. Regarding EBRT volume, the field included the compression level with a suitable margin, typically one to two vertebrae above and below the compression level. If a direct posterior field was indicated, the protocol stipulated that the prescription should be at cord depth (depth of the posterior border of the vertebral body calculated from the diagnostic MRI images). IMRT and stereotactic radiation therapy technique were not allowed. The emergency context made the implementation of central QA impossible.

After provision of informed consent and completion of EBRT simulation, patients were randomised to one of the two arms. In the control arm, patients received a total dose of 20 Gy/5Fx (4 Gy/Fx) starting on the day of simulation. In the experimental arm, patients received 10 Gy/SF, delivered on the day of simulation.

#### Assessment

Identical follow-up schedules applied to both study groups. All patients were followed up until death. The initial assessment included history and physical examination, recording of symptoms (type, duration and graduation according to trial’s related scales), documentation of the underlying malignancy and previously received treatment (including radiotherapy) and completion of the Quality of life (QoL) questionnaire.

Following treatment, patients were assessed at 1, 5 and 12 weeks, and then every 3 months until death, with a window of 5 working days allowed for the first follow-up and 7 working days thereafter. Post-treatment evaluation included survival status, documentation of ongoing medication and requirement of further EBRT, recording of symptoms and radio-induced side effects and completion of the QoL questionnaire.

The evaluation scales were (1) an in-house modified Tomita 3-point scale for mobility (1 = ‘Unaided’, 2 = ‘With walking aid’ and 3 = ‘Bed-bound’), (2), an in-house 3-point scale for bladder function (1 = ‘Continent’, 2 = ‘Incontinent’ and 3 = ‘Catheterised’), (3) the Acute and long-term radio-induced toxicity RTOG scale, (4) VAS and (5) the EORTC QLQ–C30 questionnaire.

### Outcomes

The primary outcome was the change in mobility status between baseline and 5 weeks, as evaluated by the in-house modified Tomita mobility scale.

The secondary objectives were to analyse QoL (evaluated by EORTC QLQ–C30 questionnaire, addressed in another paper^21^), radiation-induced toxicity (RTOG toxicity scale), pain control (evaluated by a visual analogue scale, addressed in another paper^21^), bladder function (evaluated by an ‘in-house’ bladder function scale) and overall survival (OS).

### Statistical analysis

Individual patient changes in mobility and bladder function scores were determined by calculating the difference between the baseline score and the score at the time of follow-up. Mean differences with 95% confidence interval (CI) in change between the groups were computed. A paired-sample *t* test was used to compare differences, from baseline to the 5-week follow-up. One-way between-group analyses of covariance (ANCOVA) were conducted to compare the effectiveness of the two different radiation schedules. For these analyses, the independent variable was the radiation schedule, the dependent variable was the score at 5 weeks post treatment and the covariate was the baseline score. Preliminary checks were conducted to ensure that there was no violation of the associated assumptions of normality, linearity, homogeneity of variances and homogeneity of regression slopes. All available data from eligible patients were included in the analyses.

Safety analyses included all eligible patients. The maximum toxicity occurring within 5 weeks of completing treatment and late toxicity—defined as events occurring or persistent 90 days or later post EBRT, were tabulated.

OS was calculated from the date of randomisation until death. Time to neurological deterioration was calculated from the date of randomisation to the date of the first worsening of either mobility or bladder function or the last follow-up. The following pre-determined potential prognostic factors for OS were evaluated: preserved baseline mobility, baseline KPS, young age, primary other than lung and dose fractionation. The Kaplan–Meier method was used to estimate OS. The log-rank test was used to compare differences in survival. The Cox proportional hazard model was used to assess potential prognostic factors on survival times.

Other continuous variables were analysed using the Mann–Whitney *U* test, and categorical variables were compared between arms using Fisher’s exact test. Statistical tests were two-sided, except for the primary endpoint, and assessed for significance at the 0.05 level. Statistical analyses were carried out using IBM^®^ SPSS^®^ statistical software version 23. All analyses were performed according to the intention-to-treat principle. The protocol did not allow the imputation of missing values.

## Results

### Patient accrual and follow-up

From January 2006 to April 2014, five Irish institutions accrued 117 patients. Five patients were found to be ineligible (one because of previous irradiation of the relevant spinal segment not documented at the time of initial assessment and inclusion, two because of a change in the pathological diagnosis at the time of post-radiotherapy review and two because of non-completion of whole-spine MRI).

Seventy-six percent of the 1466 patients screened did not meet the inclusion criteria. The main reasons were MRI-documented MSCC not fulfilling the radiological definition (45%), no MR and/or no whole-spine MR performed (9%), no available documented histological proof of malignancy at the time of initial evaluation (18%) and previous irradiation of the relevant spinal segment (8%).

**Fig. 1:**
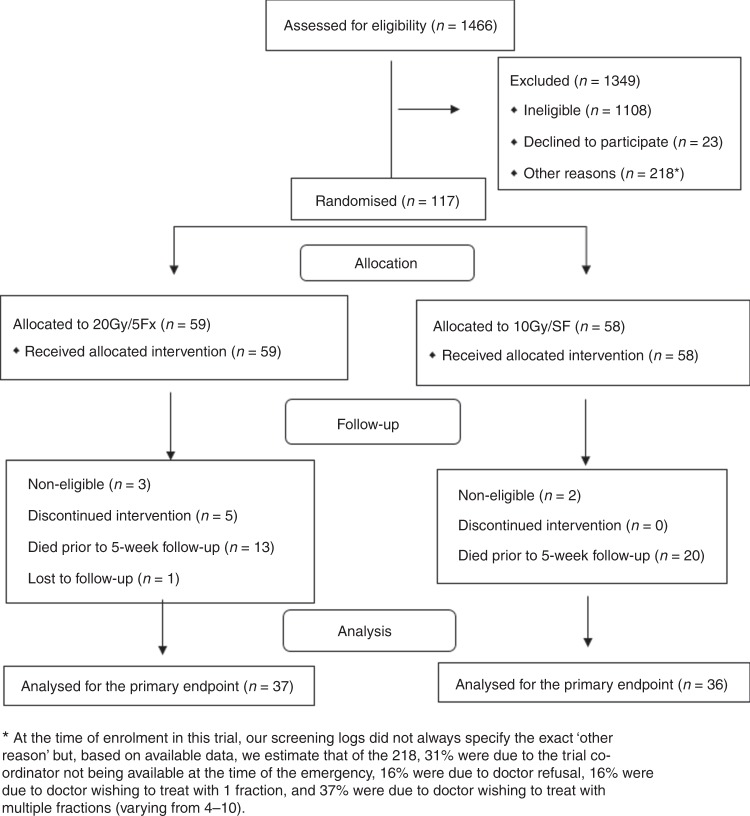
CONSORT flow diagram. ICORG 05-03 CONSORT flow diagram of two radiation schedules in malignant spinal cord compression.

Five patients in the 20 Gy/5Fx arm could not complete the allocated treatment because of early death or significant alteration of their general condition, but not because of toxicity. One-hundred patients were assessed at the 1-week follow-up. Thirty-three patients died before the 5-week follow-up, confirming that the protocol predicted a high attrition rate—estimated at 40%. On the other hand, only one patient in the 20 Gy/5Fx arm was lost to follow-up, confirming the feasibility of the trial follow-up protocol. Consequently, 73 patients were evaluable for the primary-efficacy endpoint analysis (Fig. 1). One of the 73 (control arm) did not have an assessment at the 1-week follow-up assessment. Only 52 patients were available for the 12-week follow-up (20 had died and 1 was not contactable at the time). Twenty-two percent of the 73 patients (10 in the control arm and 6 in the experimental arm) had cauda equina.

### Patient characteristics

The baseline characteristics of evaluable patients were balanced between arms (Table 1). There was no significant association between baseline mobility and arm (*p* = 0.544). The median baseline KPS was 70% (range: 30–100). Twenty percent of eligible patients had primary lung cancer compared with 10% of evaluable patients. Forty-two percent of eligible patients could walk unaided at baseline compared with 53% of evaluable patients. At baseline, all patients were on dexamethasone with a median dose of 8 mg (range: 2–16 mg).

**Table 1:** Baseline patient demographic and clinical characteristics.

	Eligible	Evaluable	20 Gy/5Fx	10 Gy/SF
Patients, No. (%)	112	73	37 (51)	36 (49)
Age,				
Median (range)	68.8 (30–87)	68.5 (30–87)	68.5 (33–87)	67.7 (30–85)
Sex, No. (%)				
Men	72 (64)	44 (60)	20 (54)	24 (67)
Women	40 (36)	79 (40)	17 (46)	12 (33)
Primary, No. (%)				
Breast	22 (20)	19 (26)	10 (27)	9 (25)
Lung	22 (20)	7 (10)	2 (5)	5 (14)
Prostate	27 (24)	22 (30)	11 (30)	11 (31)
Other	41 (37)	25 (34)	14 (38)	11 (31)
Compression level				
Cervical	4 (4)	1 (1)	0 (0)	1 (3)
Cervical–thoracic	2 (2)	2 (3)	2 (5)	0 (0)
Thoracic	75 (67)	52 (71)	25 (68)	27 (75)
Lumbar	27 (24)	15 (20)	8 (22)	7 (19)
Lumbar–sacral	1 (1)	0	0	0
Sacral	3 (3)	3 (4)	2 (5)	1 (3)
Mobility				
Unaided	47 (42)	39 (53)	18 (49)	21 (58)
With walking aid	28 (25)	16 (22)	10 (27)	6 (17)
Bed-bound	37 (33)	18 (25)	9 (24)	9 (25)
Bladder function				
Continent	82 (74)	53 (74)	25 (68)	28 (80)
Incontinent	7 (6)	6 (8)	4 (11)	2 (6)
Catheterised	22 (20)	13 (18)	8 (22)	5 (14)
Missing	1	1	0	1
KPS				
30	3 (3)	2 (3)	0 (0)	2 (6)
40	7 (6)	3 (4)	2 (5)	1 (3)
50	34 (30)	16 (22)	10 (27)	6 (17)
60–80	52 (46)	40 (55)	20 (53)	20 (56)
90–100	16 (14)	12 (16)	5 (14)	7 (20)
Dexamethasone dose				
Median	8 mg	8 mg	8 mg	8 mg
Range	2–16 mg	2–16 mg	2–16 mg	4–16 mg

Mobility: assessed by an in-house modified Tomita scale. Bladder function:assessed by an in-house scale.

*Gy* gray, *Fx* fractions, *SF* single fraction, *SD* standard deviation, *VAS* visual analogue scale.

### Primary-efficacy endpoint

The median follow-up for neurological assessment was 5.6 months (range: 1–100 months) from consent, for evaluable patients. Only evaluable patients were included in the 5-week mobility analysis. Overall, EBRT—whatever the radiation schedule—led to a mobility improvement or stabilisation at 5 weeks in 11 and 63% of patients, respectively, but did not prevent a worsening in 26% of them (Table 2). The mobility score improved for eight patients: two patients improved from score 3 to score 1, five patients improved from score 3 to score 2 and one patient improved from score 2 to score 1. Table 3 shows the mobility status at 5 weeks, and 12 weeks post EBRT compared with baseline (Supplementary Table 1 shows the mobility status at 1 week post EBRT compared with baseline).

**Table 2:** Five-week post EBRT mobility and bladder function (no comparison between arms).

	Total *n* = 73	20 Gy/5Fx *n* = 37	10 Gy/SF *n* = 36	Treatment effect 95% CI
*Mobility score*				
Improved	11.00%	10.80%	11.10%	
With walking aid to unaided	1.40%	0%	2.80%	
Bed-bound to unaided	2.70%	0%	5.60%	
Bed-bound to with walking aid	6.80%	10.80%	2.80%	
Same	63.00%	56.80%	69.40%	
Unaided	35.60%	29.70%	41.70%	
With walking aid	12.30%	13.50%	11.10%	
Bed-bound	15.10%	13.50%	16.70%	
Worse	26.00%	32.40%	19.40%	
Unaided to with walking aid	12.30%	10.80%	13.90%	
Unaided to bed-bound	5.50%	8.10%	2.80%	
With walking aid to bed-bound	8.20%	13.50%	2.80%	
Mean (SD) change from baseline^a^		–0.3 (0.78)	–0.06 (0.75)	–0.36 to 0.002
*Bladder function score*^*b*^				
Improved	7%	11%	3%	
Same	71%	64%	83%	
Worse	19%	25%	14%	
Mean (SD) change from baseline^a^		–0.22 (0.96)	–0.17 (0.71)	–0.40 to 0.001

*RT* radiotherapy; all evaluable data included.

Mobility score: 1 = ‘Unaided’, 2 = ‘With walking aid’, 3 = ‘Bed-bound’.

Bladder function: 1 = ‘Continent’, 2 = ‘Incontinent’, 3 = ‘Catheterised’.

^a^Negative values denote detrimental change.

^b^Data are missing for two patients, one in each arm.

**Table 3:** Cross-tabulation of the different mobility states at each time point compared with baseline.

	Walking unaided			Walking with an aid			Bed-bound		
Baseline	Week 1	Week 5	Week 12	Week 1	Week 5	Week 12	Week 1	Week 5	Week 12
Walking unaided	87%	67%	59%	13%	23%	21%	0%	10%	21%
Walking with an aid	13%	6%	12%	80%	56%	62%	7%	37%	25%
Bed-bound	11%	11%	20%	17%	28%	50%	72	61%	30%
Total number	38	29	23	20	23	17	14	21	12

The average change, in this case a deterioration, in the Modified Tomita score from baseline to the 5-week assessment, was −0.3 (SD 0.78) in the Control group and −0.06 (SD 0.75) in the Trial group. The estimated difference between the two arms is therefore 0.24 in favour of the Trial group (differences calculated as Trial–Control with positive values indicating better improvement for the Trial group), with a 95% CI for the differences in means of −0.12 to 0.6. The lower bound of the two-sided 95% CI is equivalent to the lower bound of a one-sided 97.5% CI. Since −0.4 is not included in the interval, there is evidence that 10 Gy/SF is non-inferior to 20 Gy/5Fx (Table 4).

**Table 4:** Baseline and 5-week mobility and bladder function scores.

		Baseline^a^		5-week		Between arms		
	*n*	Mean	SD	Mean	SD	Difference^c^	95% CI	*p*
Mobility score						0.24	–0.12 to 0.6^b^	0.077*/0.038**
20 Gy/5Fx	37	1.76	0.83	2.05	0.81			
10 Gy/SF	36	1.67	0.86	1.72	0.81			
Bladder function score						0.05	−35 to 0.45	0.487*
20 Gy/5Fx	36	1.54	0.84	1.78	0.96			
10 Gy/SF	35	1.34	0.72	1.51	0.89			

*SD* standard deviation, *CI* confidence interval, *Gy* gray, *Fx* fractions, *SF* single fraction.

^a^Higher score indicates worse mobility or bladder function.

^b^Comparability was defined by the lower bound not exceeding –0.4.

^c^Estimated difference of 10 Gy/SF minus 20 Gy/5Fx; a positive difference favours SF.

*p*: significance level resulting from the ANCOVA model, including terms for treatment, and baseline covariate. * Two-sided; ** one-sided.

The mean change in the mobility score was compared across the arms using ANCOVA where the response variable was the mobility score at 5 weeks, and the covariate was the score at baseline. There was a small-to-moderate statistically significant relationship between the pre-treatment and post-treatment mobility scores, as indicated by a partial eta-squared value of 0.33. After adjusting for pre-intervention mobility, the estimated difference in mean mobility score between the Control and Trial groups is 0.28 (95% CI −0.03 to 0.6). These estimates and confidence intervals are consistent with the hypothesis that the Trial arm is not inferior to the Control arm (lower bound of the confidence interval excludes the non-inferiority limit −0.4).

However, as the analysis of the mobility score as described in the protocol, treating this ordinal variable (with levels 1, 2 and 3) as continuous, might be considered flawed, the effect of the treatment group was explored further with the fitting of an ordinal logistic model where mobility was treated as an ordinal variable. The results were similar to those obtained from the ANCOVA analysis, with no significant differences between the two arms. The model showed that patients in the Trial arm had twice (2.16) the odds of a higher (vs. a lower) level of mobility than patients in the control arm, which supports the conclusion of non-inferiority of the Trial arm when treating the mobility score as numeric.

When categories were collapsed, the 5-week (*n* = 73) and 12-week (*n* = 52) ambulatory rates (unaided + aided) were 65% in the 20 Gy/5Fx arm and 78% in the 10 Gy/SF arm (*p* = 0.337), and 68 and 85% (*p* = 0.254), respectively.

### Secondary endpoints

#### Secondary-efficacy endpoint: bladder function preservation

Overall, EBRT—whatever the radiation schedule—led to a bladder function improvement or stabilisation in 7 and 71% of patients, respectively, but bladder function worsened in 19% of them (Table 2). Information on 5-week bladder function was missing in two patients, one in each study arm.

After adjusting for pre-intervention scores, there was no significant difference between the two radiation schedules on post-treatment bladder function scores F (1, 68) = 0.49, *p* = 0.487, partial eta squared = 0.007 (Table 4). There was a small-to-moderate statistically significant relationship between pre-treatment and post-treatment bladder function scores, as indicated by a partial eta-squared value of 0.27.

#### Secondary-efficacy endpoint: overall survival

For eligible patients (*n* = 112), the median OS was 3 months (95% CI = 1.5–4.5), with 1- and 2-year survival of 18 and 8%, respectively. Median OS was 3.0 months in each arm. On multivariate analysis, with age, mobility at baseline, mobility at week 5, preserved baseline mobility, KPS and lung primary cancer (Y/N) in the model, diagnosis of other than lung cancer was the strongest predictor of longer OS (HR: 3.0, 95% CI: 1.3–6.7, *p* = 0.011). Better mobility at week 5 (HR: 2.7, 95% CI: 1.3–5.6, *p* = 0.006), and younger age (HR: 1.03, 95% CI: 1.01–1.05, *p* = 0.006) were also predictive for longer OS.

For evaluable patients (*n* = 73), the median OS was 6.4 months (95% CI = 5.4–7.4), with 1- and 2-year survival of 27 and 12%, respectively. Median OS was 6.0 and 6.6 months, respectively, in the five and SF arms (*p* = 0.392).

#### Unplanned secondary-efficacy endpoint: time to neurological deterioration (death not an event)

For evaluable patients (*n* = 73), the median time to neurological deterioration was 3 (95% CI = 2.5–3.4), 1.7 and 3.1 months, respectively, in the 20 Gy/5Fx and 10 Gy/SF arm (*p* = 0.332).

#### Secondary endpoint: toxicity

For toxicity, the analysis included all eligible patients, having received at least one dose of RT and completed at least one post-baseline assessment. Eleven percent of patients (11/100) had grade 2 acute lower intestinal toxicity, five (11%) in the 20 Gy/5Fx arm and six (11%) in the 10 Gy/SF arm. Six percent of patients (6/100) had grade 2 acute upper intestinal toxicity, five (11%) in the 20 Gy/5Fx arm and one (2%) in the 10 Gy/SF arm. Four patients had grade 2 acute fatigue (three in the 20 Gy/5Fx arm and one in the 10 Gy/SF arm). Three patients had grade 2 acute oesophageal toxicity (two in the 20 Gy/5Fx arm and one in the 10 Gy/SF arm). Two patients had grade 2 acute skin toxicity (one in each arm). One patient (in the 10 Gy/SF arm) had grade 2 acute salivary gland toxicity.

Ten percent of patients (5/52) had grade 2 late intestinal toxicity, four (16%) in the 20 Gy/5Fx arm and one (4%) in the 10 Gy/SF arm. Two patients had grade 2 late fatigue (one in each arm). There was one grade 3 late adverse event (pain-upper thigh-hip) in the 20 Gy/5Fx arm, and no higher-grade toxicity was reported at any time point.

When baseline AEs were discounted, 12 (26%) patients in the 20 Gy/5Fx arm had a Grade 2–3 AE at any time after the start of RT compared with six (11%) patients in the 10 Gy/SF arm (*p* = 0.069).

#### Re-treatment

Of the 101 eligible patients who had at least one post-RT assessment, 11 (11%) received further radiotherapy for MSCC at the same site, and two of the 11 received treatment to more than one site (these two had initially been treated at one site). Nine patients (17%) in the 10 Gy/SF arm received re-treatment compared with two patients (4%) in the 20 Gy/5Fx arm (*p* = 0.058). However, the median follow-up for neurological assessment was 3.0 months for those not receiving re-treatment and 6.6 months for those receiving re-treatment.

Of the 73 evaluable patients, 11 patients (15%) received further radiotherapy at the same site, with a statistically significant association between re-treatment and arm (*p* = 0.024) with 9 patients (25%) in the 10 Gy/SF arm receiving re-treatment compared with 2 patients (5.4%) in the 20 Gy/5Fx arm. The median follow-up for neurological assessment was 6.3 months for those not receiving re-treatment and 6.6 months for those receiving re-treatment.

## Discussion

ICORG 05-03 is one of six published or presented prospective randomised trials trying to identify the optimal radiation schedule for patients diagnosed with MSCC treated by primary radiotherapy (Table 5).^19–25^ In the present era of evidence-based medicine, randomised trials remain essential to provide clinicians with the necessary evidence to guide their daily practice.

**Table 5:** Phase III randomised controlled trials comparing radiation schedules for MSCC.

Series [reference]	Randomised (*n*)	Design	Arms*	Primary outcome	Ambulatory rates	OS (months)	Grade 3–4 toxicity
Maranzano et al.^19^	300 (92% evaluable at 4 wks)	Equivalence	16 Gy/2Fx vs. split: 15 Gy/3Fx + 15 Gy/5Fx	Response rate at 4 wks	68% 71% (NS)	4 4 (NS)	6% 8% (NS)
Maranzano et al.^21^	327 (93% evaluable at 4 wks)	Equivalence	8 Gy/1Fx vs. 16 Gy/2Fx	Symptom control at 4 wks	62% 69% (NS)	4 4 (NS)	0% 0% (NS)
Abu-Hegazy and Wahba^22^	285	Unavailable	8 Gy/1Fx, 30 Gy/10Fx vs. 40 Gy/20Fx	Functional outcome at 4 wks	65.8% 66.7% 65.7% (NS)	n/a n/a n/a	0% 0% 0% (NS)
SCORE-2 Trial^23^	203 (76% evaluable at 4 wks)	Non-inferiority	20 Gy/5Fx vs. 30 Gy/10Fx	Motor function at 4 wks Wk1: 87.2% Wk1: 89.6%	71.8% 74.0% (NS)	3.2 3.7 (NS)	0% 0% (NS)
SCORAD III Tria^24^	688 (49% evaluable at 8 wks)	Non-inferiority	8 Gy/1Fx vs. 20 Gy/5Fx	Ambulatory at 8 wks	69.5% 73.3% (NS)	2.9 3.2 (NS)	20.6% 20.4% (NS)
This trial	112 (65% assessable at 5 wks)	Non-inferiority	10 Gy/1Fx vs. 20 Gy/5Fx	Change in mobility at 5 wks	78% 65% (NS)	6.6* 6.0* (NS)	0% 4% (NS)

*OS* overall survival, *Fx* fractions, *S* significant, *NS* non-significant, *wk* week.

*Evaluable patient.

Interestingly, the rationale of all these randomised trials was based on the recognition of the burden of protracted radiation schedules for patients treated with MSCC, and they all compared a prolonged schedule with an accelerated one. The trials were designed to demonstrate either equivalence^19,22^ or non-inferiority^23,24^ (design information unavailable for one trial^25^). All trials excluded patients eligible for surgery, recognising the superiority of the multimodality treatment for the very small fraction of patients eligible for surgery.^8^

Three trials^19,22,23^ included only patients with a predicted limited life expectancy (≤6 months), estimated using various algorithms. This trial did not select patients for their predicted life expectancy.

The challenge of conducting trials in the studied population is the high attrition rate related to early death reported in other trials from 7%^22^ to 24%^23^ at 4 weeks, 51%^24^ at 8 weeks post treatment and 35% at 5 weeks in this trial.

The other randomised trials compared various radiation schedules, e.g. 30 Gy delivered in 3–10 fractions,^19,23,25^ 20 Gy in 5 fractions,^20,21,23,24^ 16 Gy in 2 fractions,^19,22^ 8 or 10 Gy in 1 fraction^20–22,24,25^ and 20 × 2 Gy,^25^ illustrating the current absence of standard of care. The primary endpoints were functional outcome/mobility, assessed by various mobility scales, comparing for each individual patient the early post-treatment score achieved at 4–8 weeks with the pre-radiotherapy baseline one. All trials concluded that there was no statistically significant difference in the short-term efficacy of the short-radiation schedule when compared with the more protracted higher-dose one, with similar toxicity.

Like other trials, the present one has several limitations. The rationale and form of the non-inferiority power calculation used for the sample size estimation assumed that the outcome variable (modified Tomita score) was a continuously distributed variable that can be analysed by ANCOVA. Strictly speaking, this is not true because the outcome variable is actually a 3-point ordinal variable. So, we have to assume (1) that the outcome is effectively continuous but is ‘rounded’ to one of only three possible values, and (2) the middle option lies approximately midway between the other two options. Further analysis, fitting an ordinal logistic model where the primary outcome mobility was treated as an ordinal variable, showed results similar to those from the ANCOVA analysis, with no significant differences between the two treatment arms. The model showed that patients in the Trial arm had twice the odds of a higher level of mobility than patients in the control arm, which supports the conclusion of non-inferiority of the Trial arm when treating the mobility score as numeric.

Our study was powered to find as unacceptable a detrimental difference of −0.4 between the two arms in the mobility-scale change between the baseline and 5-week assessment. The administrative error whereby three patients were identified as non-eligible after the closure of the study, had minimal effect, as the confidence interval for the differences between the groups is clearly above the −0.4 non-inferiority limit, so there is evidence of non-inferiority.

In addition, the steroids and the EBRT technique (field arrangement, beam type and energy) were left to institutional practice and physician preferences, partly due to the context of emergency. The applied radiotherapy technique therefore varied, with the sole restriction being the exclusion of IMRT and stereotactic-ablative radiotherapy.

Finally, the evaluation of the long-term local efficacy of the compared radiation schedules is made difficult because of the limited life expectancy of patients diagnosed with MSCC. In this trial, there was a significant difference in the KPS between those eligible and evaluable at the 5-week follow-up, suggesting that the KPS eligibility criterion was perhaps too low. Eligible patients had a median KPS of 50% compared with a median of 70% for evaluable patients (*p* = 0.001).

The potential limitation of short-course radiotherapy is a higher risk of in-field recurrence. Such a risk has been suggested in various publications. In a prospective non-randomised study, Rades et al. found that long-course RT resulted in significantly better 1-year in-field local control;^26^ however, the same authors did not find any difference for patients with limited life expectancy.^27^

Three of the randomised trials also reported the in-field recurrence rate. Abu-Hegazy et al.^25^ reported a statistically significantly higher cumulative rate of in-field recurrence at 2 years in patients treated with a SF of 8 Gy, when compared with those treated with protracted schedules [2-year in-field recurrence rate: 22.2% (8 Gy/1) vs. 16.1% (30 Gy/10) and 13.5% (40 Gy/20), *p* = 0.01]. Using MRI-based diagnosis, Maranzano et al. reported a higher in-field recurrence with lower dose short schedules in both their trials (3.5% (16 Gy/2) vs. 0% (30 Gy/8)^19,22^ and 6% (8 Gy/1) vs. 4% (16 Gy/2)^22^) with a median time to occurrence of 5–8 months. In the present trial, the same was observed, with an increased rate of re-irradiation in patients on the SF arm, and median time to occurrence of less than 3 months.

The six randomised trials confirm the conclusions of the George et al. Cochrane review,^28^ which recommended a short-radiation schedule for ambulant adults with metastatic extradural MSCC with stable spines, and predicted survival of less than 6 months. When this trial was designed, the predicted survival score, generated by Rades et al. based on data from 1852 spinal cord compression patients, was not available.^29^

However, more research is warranted to improve the outcome of patients with MSCC treated with primary radiotherapy. As demonstrated by the published evidence, patient selection is crucial, and refinement of currently available individualised prognosis prediction tools is necessary to allow personalised management plans. The observed high early death rate—within the first 2 months after completion of radiotherapy—supports the hypothesis that some patients will not benefit from primary radiotherapy. However, the higher rate of in-field recurrence associated with short-course radiotherapy in patients surviving beyond 6 months provides a rationale for more aggressive therapy to improve local control. Re-treatment was addressed by Rades et al. in a retrospective investigation of 124 patients with rather favourable results.^30^ Radiation myelopathy was not observed after re-treatment. Thus, re-treatment appears feasible and helpful.

It should be noted that the response rate calculation method can be misleading, as it usually includes both ambulatory patients maintaining mobility and non-ambulatory patients recovering mobility. Unfortunately, the reported ambulatory recovery rate remains largely below 50%. Beyond demonstrating the importance of early diagnosis and treatment, this also highlights the limitation of EBRT.

One potential avenue of improvement is the use of a radiation-ablative schedule as this has demonstrated benefit in other clinical scenarios. The results of this trial indirectly support the rationale of such an approach, as an unplanned analysis showed a trend in favour of the SF schedule when the results are reported by preserved ambulatory rate-collapsed categories. Promising results coming from limited institutional experience and small phase II trials of Stereotactic Body Radiation Therapy or Radiosurgery in patients with MSCC have been reported. Ryu reported a 52% recovery rate and a 11% improvement rate in patients (*n* = 27) with symptomatic MSCC treated with radiosurgery delivering a SF of 16 Gy (12–20 Gy).^31^ Other authors reported similar promising outcomes with low toxicity,^32–34^ indicating that spine radiosurgery has the potential to change clinical practice in the management of MSCC.

This randomised trial demonstrates that 5-week mobility in the experimental arm, 10 Gy/SF, was non-inferior when compared with the multi-fraction standard arm, 20 Gy/5Fx. Given the convenience of the single-fraction radiation schedule, both for patients and health facilities, the SF schedule should be considered in patients diagnosed with MSCC with a predicted short life expectancy.

## Supplementary information

Supplementary Table 1

## Data Availability

Data supporting the results reported in this article can be requested from Cancer Trials Ireland.
