# Supplementary material for: Non-inferiority randomised phase 3 trial comparing two radiation schedules (single vs. five fractions) in malignant spinal cord compression
Source: Br J Cancer. 2020 Mar 11;122(9):1315–23. doi: 10.1038/s41416-020-0768-z (PMC7188681; doi:10.1038/s41416-020-0768-z)
Supplement: Supplementary file 1 — Supplementary Table 1 [file 41416_2020_768_MOESM1_ESM.docx]

**Supplementary Table 1: 1-week post EBRT Mobility function (no comparison between arms)**

|  |  | Total | 20Gy/5Fx | 10Gy/SF | Treatment effect 95% CI |
| --- | --- | --- | --- | --- | --- |
|  |  |  | N= 46 | N= 54 | N= 100 |
| Mobility score | Improved  Same  Worse | 10%  79%  11% | 6.5%  78%  15% | 13%  80%  7% |  |
|  | Mean (SD) change from baseline* |  | - 0·09 (·46) | 0·06 (.66) | -0·12 to 0·10 |

Abbreviations: RT: Radiotherapy; All evaluable data included.

Mobility score: 1= ‘Unaided’, 2= ‘With walking aid’, 3= ‘Bed-bound.

*: negative values denote detrimental change.
